# Glasshouse Evaluation of the Black Soldier Fly Waste Product HexaFrass™ as an Organic Fertilizer

**DOI:** 10.3390/insects12110977

**Published:** 2021-10-28

**Authors:** Sara Borkent, Simon Hodge

**Affiliations:** School of Agriculture & Food Science, University College Dublin, D04 V1W8 Dublin, Ireland; simon.hodge@ucd.ie

**Keywords:** black soldier fly, *Hermetia illucens*, frass, circular economy, organic agriculture

## Abstract

**Simple Summary:**

In recent years farmers have relied on highly efficient synthetic nitrogen fertilizers to achieve increased yields. However, the extensive application of nitrogen-based fertilizers is now associated with several negative impacts on the environment, such as pollution of waterways and eutrophication of lakes and estuaries. To promote more sustainable food production, less environmentally damaging methods of adding nutrients and organic matter to soils are needed. One potential organic fertilizer has arisen recently as a by-product of insect farming. Mass production of the black soldier fly (BSF; *Hermetia illucens* L.) results in the production of waste or frass, which is high in organic matter and contains essential plant nutrients such as nitrogen, phosphorous and potassium. In this study, it was found that one such product, HexaFrass™, improved the shoot and root growth of several herb and vegetable plants when grown under glasshouse conditions, and had similar effects to other commonly-used organic fertilizers, such as chicken manure pellets. As HexaFrass™ is a waste by-product, and the BSF are themselves raised on other food or organic wastes, the use of BSF frass has good potential as a sustainable, more environmentally-friendly, organic soil amendment.

**Abstract:**

The mass farming of the black soldier fly (BSF; *Hermetia illucens* L.), to produce insect-based feed for livestock and fish, results in considerable amounts of insect frass, which contains substantial amounts of organic matter and bioavailable nutrients. Insect frass has shown good potential as a soil amendment and organic fertilizer. This study examined the effects of HexaFrass™ on the growth of common vegetables and herbs under glasshouse conditions. In an organically-rich potting mix, HexaFrass™ increased shoot dry weight by an average of 25%, although this effect was variable among test plants. In other trials, application of HexaFrass™ caused an increase in plant growth similar to that obtained by applying chicken manure and a commercial organic fertilizer. Increases in shoot and root dry weight showed quadratic relationships with HexaFrass™ dose, indicating that application of excessive quantities could lead to plant inhibition. Shoot:root dry matter ratio tended to increase with HexaFrass™ dose suggesting there was no specific stimulation or enhancement of root growth. Overall, these results provide further evidence of the potential of insect frass as an effective organic fertilizer for vegetables and herbs.

## 1. Introduction

The agricultural sector is under constant pressure to produce increasingly more food due to the growing global population [1]. Historically, farmers have relied on area expansion and use of highly efficient synthetic nitrogen fertilizers to achieve increased yields, however these methods are no longer considered viable or sustainable in the long term. Expansion of agricultural land comes at the cost of habitat and biodiversity loss, whereas the application of synthetic nitrogen compounds and the use of biocides required to intensify production is associated with a suite of negative environmental consequences [1,2,3].

There is, therefore, a growing realization that to promote a more sustainable intensification of food production, less environmentally damaging methods of adding nutrients and organic matter to soils need to be explored. One relatively recent source of organic fertilizer has arisen as a by-product of mass production of the black soldier fly (BSF; *Hermetia illucens* L.) as a source of protein. This dipteran species is one of the most commonly reared insects for the production of protein feed for livestock, primarily because it has a short life cycle, has a high feed conversion efficiency, and can be reared on a large variety of waste products as a substrate [4]. The BSF larvae have a high crude protein and oil content, making them an ideal source of protein and fats for other livestock [5,6].

When compared with more conventional sources of protein for animal feeds, such as soy and fishmeal, the production process of BSF protein is more sustainable environmentally and economically [7,8]. Using BSF waste as a fertilizer would contribute to the transition towards a more circular economy in the agricultural sector in several ways. Firstly, the utilisation of a waste product as fertilizer would reduce the need to produce nitrogen fertilizers from other resources [8]. Secondly, since BSF frass is a by-product of another process, its production does not bring any further waste into the system. Thirdly, the production of the BSF larvae themselves is based on producing valuable protein feed by rearing larvae on waste from another process.

As a by-product, the BSF frass (BSFF) consists of a mixture of leftover feed material, insect skins and faeces, and microorganisms [7,8]. In recent years, there have been a number of studies exploring the potential of frass as a fertilizer, and several reports of how BSFF can have positive effects on plant growth, yield, soil structure and the soil microbiome [8,9,10,11,12,13]. BSFF may also provide additional benefits such as increasing soil organic matter and supplying micronutrients such as copper and zinc, that are essential for plant growth and development [8,13].

Nevertheless, several aspects of the efficacy of BSFF as a plant growth promoter require clarification. For example, variability in the effects of BSFF on plant performance can occur due to differences in the larval diet [11] and variation in climatic conditions [14]. Other studies have indicated there may be an “optimum” soil concentration of BSFF, and that once this concentration is exceeded plant growth may be inhibited [10,12]. This variability indicates that the results obtained from different sources of BSFF, and results obtained under different environmental conditions, may not be directly transferrable to new products or different growing situations. Thus, before wider generalizations of BSFF effectiveness can be inferred, and the product can justifiably be sanctioned as part of a circular economy, the efficacy of each new source of material must be assessed, ideally under a range of growing conditions.

The BSFF product HexaFrass™ (HF) is produced by the company HexaFly in County Meath, Ireland, via the mass-rearing of BSF primarily on brewery waste. HF is certified organic and described as a slow-release fertilizer which contains approximately 60% organic matter, including some protein and chitin. Publicity material suggests HF may boost soil ‘health’, stimulate strong root growth, and induce resistance against insect pests such as aphids (hexafly.com). Although we could find no published literature or available data on the functioning of this product, HF is now widely available in commercial agricultural and gardening outlets in Ireland.

There is some ambiguity over when organically-derived products should be described as fertilizers, soil amendments, biostimulants or plant protection agents. These decisions are generally based on whether effects are caused by direct nutrient supply, or via some biostimulant effect that indirectly improves plant nutrient uptake, stress tolerance, or resistance to challenge by pests and pathogens [15]. Additionally, the impacts of plant growth promoters can be highly variable, and positive effects sometimes only transpire under narrow environmental conditions, or when plants are experiencing biotic or abiotic stresses [16,17,18,19,20,21,22]. Therefore, it is generally necessary to examine the responses of several plant species, under a range of growing conditions, in order to make meaningful generalizations concerning the effectiveness of new products [23,24].

The aims of this research were to gain preliminary information regarding the effects of HF on seedling growth under glasshouse conditions. We examined the growth responses of eleven common garden flowers, vegetables, and herbs to application of HF as a soil amendment, and compared the effects of HF on shoot and root growth when using potting media with different organic contents and different basal nutrient levels. Finally, we also examined the effect of applying different quantities of HF, and whether adding HF to soil in a single application or as a split application, produced different effects on plant growth.

## 2. Materials and Methods

### 2.1. General Methods

All experiments were performed in glasshouses at University College Dublin Rosemount Environmental Research Station between May and August 2021. During this period mean temperature in the glasshouse was 24.5 °C with a standard deviation of 8.3, and the mean relative humidity was 54% with a standard deviation of 18.

The plants used in these trials were: basil (*Ocimum basilicum* L. “Cinnamon”), borage (*Borago officinalis* L.), buckwheat (*Fagopyrum esculentum* Moench), cabbage (*Brassica oleracea* L. “Deadon F1”), celery (*Apium graveolens* L. “Victoria F1”), chicory (*Cichorium intybus* L. “Rossa di Treviso”), hyssop (*Hyssopus officinalis* L.), lettuce (*Lactuca sativa* L. “Blonde de Paris”), parsley (*Petroselinum crispum* (Mill.) Fuss “Neapolitanum”), phacelia (*Phacelia secunda* J.F.Gmel.), sage (*Salvia officinalis* L.). All seeds were purchased from The Organic Centre, Leitrim, Ireland, or Quickcrop, Sligo, Ireland.

All assays were carried out using 7 × 7 cm plastic pots as the independent experimental unit. Each pot contained an individual seedling, and were placed onto individual plastic trays to prevent nutrient leaching and cross-contamination of treatments. Plants were watered every 2–3 days using untreated water. At harvest, shoots were removed, placed into paper bags, and dried in an oven at 65 °C for 3 d. Where roots were also harvested, these were washed to remove residual soil, placed into paper bags, and dried as per the shoots.

The test substance HexaFrass™ (HF) was obtained direct from Hexafly, Meath, Ireland. HF is stated to have an organic matter content of 60%, an NPK of approximately 3-2-1, and contains the biopolymer chitin (hexafly.com). Of the 3% total nitrogen, 2.3% is described as insoluble or slow-release, and 0.7% as soluble N and fast-release. Additionally, analysis by HexaFly has indicated HF contains other important plants nutrients, such as sulphur (6 g/kg), magnesium (5 g/kg), iron (300 mg/kg) and copper (12 mg/kg) (Alvan Hunt personal communication, 19 October 2021).

Two commercial organic fertilizers were included in trials to act as positive controls: Westland Organic Chicken Manure Pellets (chicken manure; CM) with an NPK of 4.5-3.5-2.5, and Miracle-Gro Performance Organics All Purpose Fertilizer, a granular organic fertilizer (OF) with an NPK of 4-1.5-4). To emulate a top dressing, the fertilizers were applied in dried form to the surface of each pot, and then gently forked into the growing medium before being watered in. 

### 2.2. Effects of HexaFrass™ in Growing Media with High Organic Content

Ten plants were used in this trial, consisting of four herbs (basil, hyssop, parsley, sage), three vegetables (cabbage, celery, chicory) and three flowering plants commonly used to attract and sustain pollinating insects (borage, buckwheat, phacelia). Seeds were direct sown into a 1:1 mix of Westland Nutrient Rich Garden Soil and Westland Multi-Purpose Compost, and thinned down to one plant per pot at the cotyledon stage.

The recommended rate for HF for vegetables is ‘25 g per 1 square foot’ which, for simplicity, can be rounded to approximately 1.5 g HF per 7 cm square pot. Thus, in this trial HF was applied at a standard rate (1.5 g per pot) and a high rate (2 × 1.5 g per pot) with the second application added to the pots between 8 and 13 days after the first. CM was applied at the same time of the first HF application at a rate of 1.5 g per pot to act as a positive control.

Because the test plants grew and developed at different rates, fertilizer treatments were applied, and the plants harvested, after different time intervals (Supplementary Table S1). For example, for fast growing plants such as borage and buckwheat the first HF and CM application occurred 16 d after sowing, and plants were harvested after 37 d. For slower growing species, such as celery and sage, the first fertilizer application occurred after 28 d and the plants were harvested after 54 d (Supplementary Table S1).

Each of the ten plant species had five replicates of each of the three fertilizer treatments plus five replicates of a ‘no fertilizer’ control. Thus, there were 200 plants used in total arranged in a completely random design. At harvest, only the above-ground parts were retained, and growth measured as shoot dry weight.

### 2.3. Comparison of the Effects of HexaFrass^TM^ in High and Low Nutrient Potting Mix

The plant species used in this experiment were lettuce, basil, and parsley. Seeds were sown into trays and transplanted into individual pots at the two-leaf stage. Plants were grown in two potting mixes, representing relatively high and relatively low nutrient substrates. The high nutrient mix consisted of equal parts Westland Nutrient Rich Garden Soil, Westland John Innes No.2, and Westland Composted Bark. The low nutrient mix consisted of equal parts Westland Composted Bark, horticultural sand, and Coco & Coir Coco Grow, which is a nutrient free coconut coir growing medium.

Fertilizer treatments were applied 1-week after transplanting, and consisted of 2 g HF, 2 g CM and 2 g OF, along with a no-fertilizer control. There were four replicate plants of each fertilizer treatment in each growing media arranged in a randomized block design. Plant growth was quantified using shoot dry weight.

### 2.4. The Effect of Applying HexaFrass^TM^ in a Single Application versus Split Application

This assay used lettuce, basil and parsley as test plants and the high and low nutrient potting mixes described above. HF was applied to pots 1 week after transplanting the seedlings at the rates of 0 g and 4 g. An additional treatment consisted of a split application where 1 g HF was applied four times, separated each time by a 1-week interval. There were four replicates of each of the three treatments, for each plant species, in each growing medium. Plant growth was quantified using shoot dry weight.

### 2.5. The Effect of HexaFrass^TM^ Dose on Plant Growth and Shoot:Root Ratio

This assay used lettuce, basil and parsley as test plants and the high and low nutrient potting mixes described above. HF was applied to pots 1-week after transplanting at the rates of 0, 1, 2, 4 and 8 g per pot. There were four replicates of each treatment, for each plant species, in each growing medium. Plant growth was quantified using shoot and root dry weights, and these values were then used to calculate a shoot:root dry weight ratio for each plant.

### 2.6. Data Analysis

All data were collated and manipulated using Microsoft Excel, and statistical analyses were performed using Genstat v 19 (VSN International, Hemel Hempstead, UK). For the trial using the high organic content potting mix, each plant species was initially considered separately, and the shoot dry weights of plants in the four treatments compared using one-way ANOVA. Treatments were compared in a pairwise fashion using unprotected least significant differences (LSDs; *p* < 0.05). To evaluate the overall effects of the three fertilizer treatments over all ten plant species, a residual maximum likelihood (REML) analysis was performed using data from all plants, with fertilizer treatment included as a fixed factor and plant species included as a random factor.

To evaluate the effects of HF in the high and low nutrient potting mix, each plant species was considered separately, and general linear models were performed with shoot dry weight as the response variable and soil type and fertilizer treatment as explanatory factors. Pairwise comparisons of treatments were performed using the LSD.

To evaluate the effect of HF dose on shoot and root dry weight, and shoot:root dry weight ratio, the data for each plant species was considered separately. Initially, polynomial regression curves were fitted for each response variable using a quadratic model of the form *y* = *a* + *b* (HF) + *c* (HF^2^). If the second term (HF^2^) was not statistically significant, then this term was dropped, and a linear regression model fitted. If the linear model was not significant then no regression line was fitted.

For the polynomial relationships, HF_Max_, the HF dose that would produce the maximum dry weight, was calculated as −*b*/2*c*. HF_Max_ was then substituted into the appropriate polynomial equation to obtain the maximum value of the response variable that would be obtained by adding this amount of HF.

## 3. Results

### 3.1. Effects of HexaFrass^TM^ in Growing Media with High Organic Content

In the trials using the nutrient rich compost mix, the average shoot growth of eight of the ten test plants was positively affected by the application of HF, although this difference was found to be statistically significant for only two plants, basil, and phacelia (Table 1). Additionally, there appeared no consistent effect of adding 3 g HF compared with adding 1.5 g HF. However, when the data from all plants were considered in the same REML analysis, there was a significant overall 24.2% increase in shoot growth when 1.5 g HF was added to each pot and even higher 29.5% average increase when 3 g HF was added to each pot (Table 1).

In terms of assessing the effects of the positive control, adding 1.5 g CM to each pot resulted in similar effects to HF. In seven of ten plant species, the chicken manure caused an increase in average shoot growth compared to the control, but this was only statistically significant (based on LSD values) for borage (Table 1). However, taken over all the ten plant species, the REML analysis indicated the CM caused a significant increase in average shoot dry weight of 22.9% compared with that obtained in the no-fertilizer control plants (Table 1).

### 3.2. Comparison of the Effects of HexaFrass^TM^ in High and Low Nutrient Potting Mix

The application of 2 g HF per pot increased the average shoot dry weight for all three plants in both potting mixes, with increases ranging from 6× to 34× the dry weight obtained in the no-fertilizer controls (Table 2). HF significantly increased the shoot dry weight of basil in the low and high nutrient soils, and the shoot dry weight of lettuce and parsley in the high nutrient potting mix (Table 2). HF also increased the average shoot dry weight of lettuce and parsley in the low nutrient potting mix by over ten-fold, but these differences were not statistically significant. In terms of the other fertilizers, the 2 g HF treatment had similar effects on shoot dry weight as CM and OF for all three plants in both potting mixes, and HF was the by far the superior fertilizer for parsley in the high nutrient potting mix (Table 2).

The parsley seedlings did not perform well in the low nutrient potting mix, which caused low growth, highly variable data, and low survival in the OF treatment, where only one of the four plants survived to harvest (Table 2). This resulted in this combination of plant species and potting mix being the only occasion where we did not find a statistically significant difference among treatments.

### 3.3. The Effect of Applying HexaFrass^TM^ in a Single versus Split Application

**Figure 1:**
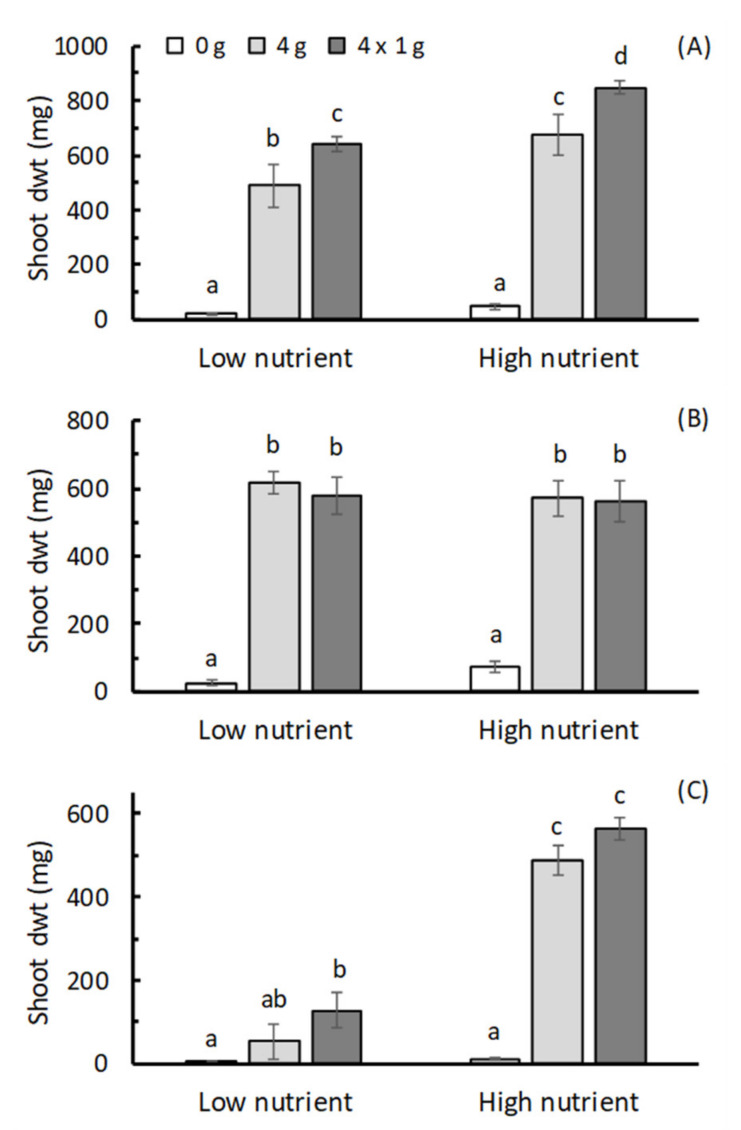
Effects of adding HexaFrass™ at rates of 0 g, 4 g and a 4 × 1 g split application on the shoot dry wt (mean ± se; n = 4) of (**A**) basil, (**B**) lettuce and (**C**) parsley seedlings in low and high nutrient potting mix. For each plant, treatments sharing the same letter code deemed not to be significantly different using LSD obtained from ANOVA at *p* < 0.05.

For all the three plants there was a significant effect of fertilizer treatment on shoot dry weight (*p* < 0.001; Supplemental Table S2), with both the 4 g HF and 4 × 1 g HF treatments generally increasing dry weight compared with that achieved in the no-fertilizer control (Figure 1). The exception to this was for parsley grown in the low nutrient potting mix, where only the 4 × 1 g split HF application was separated from the control in terms of dry weight.

When comparing the 4 g single HF application and 4 × 1 g split HF application, there was no significant difference in shoot weight for lettuce and parsley in both high and low nutrient soils (Figure 1). In basil, however, the 4 × 1 g split application produced significantly higher shoot dry weight in both potting mixes (Figure 1).

### 3.4. The Effect of HexaFrass^TM^ Concentration on Plant Growth and Shoot:Root Ratio

**Figure 2:**
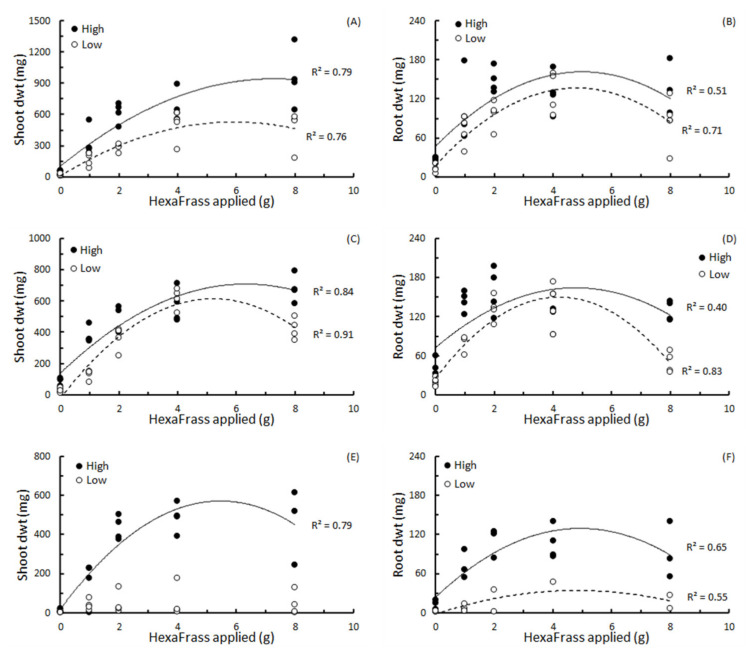
The relationships between shoot and root dry weight and amount of HexaFrass™ (g) applied to high or low nutrient potting mix for (**A**,**B**) basil, (**C**,**D**) lettuce and (**E**,**F**) parsley. R^2^ values produced from fitting polynomial order 2 regression curves.

Generally, shoot and root dry weight showed quadratic relationships with the amount of HF added to each pot (Figure 2; Table 3). The only exception to this was the parsley grown in low nutrient potting mix where no discernible relationship between shoot dry weight and HF could be established (Figure 2E; Table 3).

**Figure 3:**
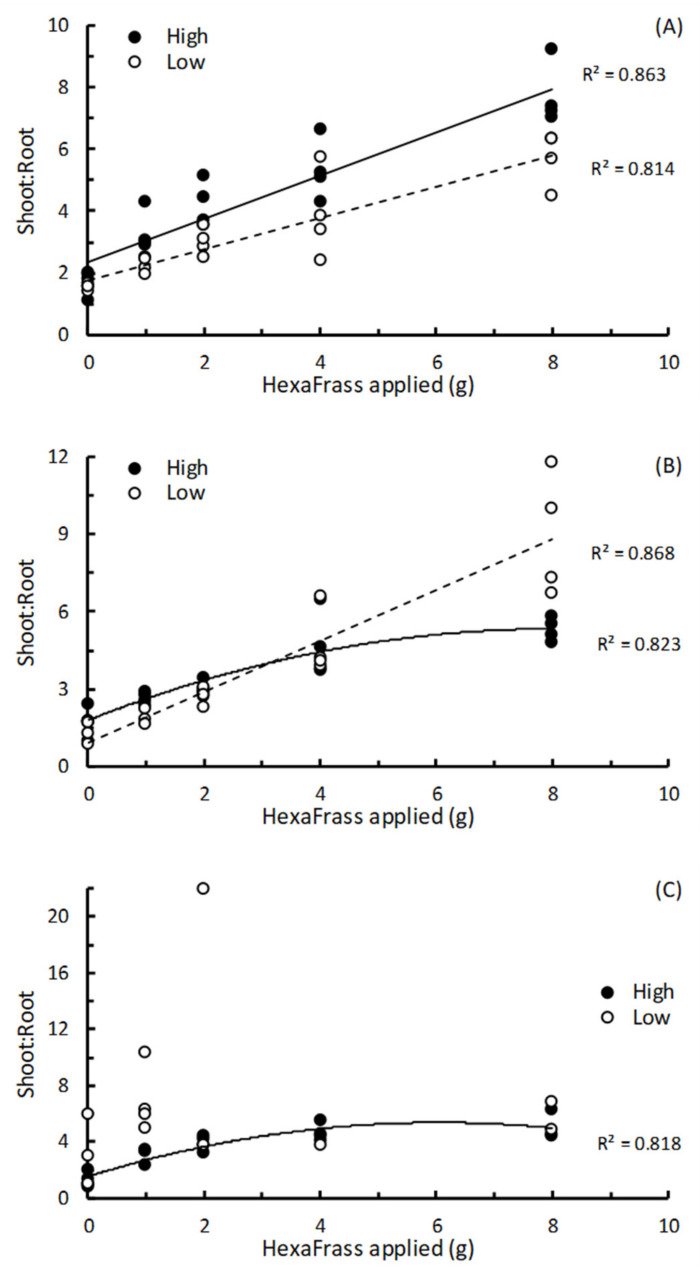
The relationships between shoot:root dry weight ratio and amount of HexaFrass™ (g) applied to high or low nutrient potting mix for (**A**) basil, (**B**) lettuce and (**C**) parsley. R^2^ values produced from either linear regression or fitting polynomial order 2 regression curves.

For basil in both high and low nutrient potting media, and lettuce in the low nutrient potting mix, the shoot:root dry weight ratio showed linear relationships with HF dose, indicating that effects of additional HF on shoot growth were exceeding that of root growth (Figure 3A,B; Table 3). For lettuce and parsley in the high nutrient potting mix, the relationship between shoot:root ratio and HF dose was quadratic, indicating that at low HF doses the effect of HF on shoot growth exceeded that seen in the roots, but as the HF dose was increased this differential between shoot and root dry weight was beginning to close (Figure 3B,C; Table 3). No discernible relationship between the shoot:root ratio of parsley grown in low nutrient potting mix with HF dose could be established (Figure 3C; Table 3).

In terms of the ‘optimum’ amount of HF to add to each pot to achieve the highest shoot or root dry weight, this ranged from 4.18 g for lettuce roots in low nutrient potting mix, to 7.31 g for basil shoots in the high nutrient potting mix (Table 3). Overall, all the HF_Max_ values were greater than 4 g per pot, and all the HFMax values for shoots were higher than the HF_Max_ values for roots for the same plants in the same soils (Table 3).

## 4. Discussion

Viewed over all the various assays, HexaFrass™ generally had a positive effect on plant growth. To varying degrees, application of HF to seedlings as a top-dressing increased shoot dry weight of seedlings in an organically-rich compost based growing medium, and two potting mixes based on garden soil, bark chips, and coir fibre. These results reinforce findings of other studies that similarly demonstrated increases in plant growth in response to application of BSFF-based fertilizers [8,9,10,12]. HF also produced similar increases in shoot growth to two other standard fertilizers, chicken manure pellets and a commercial organic fertilizer [9,13].

When using the organically rich potting mix, although HF caused an overall increase in shoot growth of around 25–30%, only two of the ten plants showed statistically significant responses to HF when considered individually. When using this growing medium it was unlikely the plants were experiencing any significant nutrient deficits given the relatively short duration of the trials, so the addition of additional nutrients was possibly only ever likely to induce minor gains [21,22]. Most of the plant species (8/10) tested did show a positive response to HF, but the combination of low replication and some high variation within treatments meant these differences were not identified as statistically significant. These results highlight the need to consider the statistical power of similar plant growth assays when trying to detect small effects. Where feasible, statistical power can be enhanced by increasing replicate numbers, or by pooling data from multiple assays and adopting a meta-analysis approach [22,24], (Supplementary Figure S3).

In the trials using the two potting mixes assigned as high and low nutrient contents, we found that the addition of HF, and the other fertilizers, generally increased growth in both growing media. This was unexpected, as we had presumed that the effects seen on plant growth by adding fertilizer products to the low nutrient medium would be greater than in the high nutrient medium [21,22]. Although we did not analyze the potting media for nutrient levels, comparison of control plants clearly demonstrated increases in shoot and root growth in the high nutrient potting mix compared with that obtained in the low nutrient potting mix (Supplementary Figure S4). Nevertheless, in retrospect, it would appear that both of these potting mixes were actually sub-optimal in terms of providing adequate nutrition for healthy plant growth. The results obtained when using the organically rich potting mix probably provide the best indication of how the effectiveness of HF might be reduced when the majority of the plant’s nutrient requirements are supplied by the growing media.

In the trial high organic potting mix, the high HF dose (3 g per pot) caused a slight, but statistically significant, higher increase in overall plant growth than the low HF dose (1.5 g per pot; 29.5% *v* 24.2%). In the other potting mixes, the relationships between HF application rate and shoot and root growth tended to be quadratic, indicating there was quantity of HF that would ‘optimize’ plant performance, and that excessive HF could inhibit plant growth. In our experimental system, highest shoot and root growth of test plants occurred when HF was applied at over 4 g, but less than 8 g, per pot, in the region of 1–2% soil volume. Other studies using lettuce and pak choi [10,12] have also reported stunting effects on plant growth when BSFF was applied at high volumes. However, these previous studies applied BSFF at rates in the region of 30–50% soil volume, far higher than the maximum rate of HF used in our study (8 g HF ≈ 2% total soil volume). So, although patterns showing the non-linear relationship between plant growth and BSFF are similar among studies, these results provide additional evidence that results using different BSFF products may not be directly comparable [8,11]. Previous studies have highlighted that the quality or effectiveness of BSFF as a fertilizer can be influenced by the diet or substrate on which the BSFF larvae are raised [11]. For commercial operations, these findings suggest that if methods of producing BSF larvae are revised, the properties of the frass bi-product might also be modified. Additionally, direct comparison of the effectiveness of multiple BSFF products would be highly valuable in relating responses in plant growth to the nutrient, chemical and physical properties of the frass.

At low doses (≤4 g per pot), application of HF increased root growth, although at the highest dose we tested (8 g HF per pot) root dry weight had either plateaued or started to decrease. However, the shoot:root dry weight ratio showed a linear increase in basil, and in lettuce in the low nutrient potting mix. For lettuce and parsley in the high nutrient potting mix, the shoot:root ratio showed quadratic relationships with HF dose, and, finally, for parsley in low nutrient potting mix no pattern could be discerned. This latter result transpired because the parsley plants did not perform well in this medium, and produced some spuriously high shoot:root ratio because only minute amounts of root material could be retrieved. These results corroborate general findings where shoot:root ratios increase with increased nitrogen supply, which results in a disproportionate growth of the shoot compared with root [25]. So, although HF increases root dry matter, this appears to be more in line with an overall increase in total plant growth, and not a specific enhancement or stimulation of root growth. There is often a correlation between shoot:root ratio and leaf protein content, however, so these results may suggest that HF not only causes an increase in shoot dry weight but also in the nutritional quality of plant foliage [25,26]. This effect would be of high relevance to the quality of pasture and mixed species swards grown for livestock grazing and requires further investigation and clarification.

When applying 4 g of HF in a single application or as four 1 g applications, the three test plants produced different results, with basil responding positively to split application, lettuce showing no difference, and parsley being intermediate. There is an obvious trade-off between the extra labour involved with split applications and any additional growth obtained. The 4 g HF we used in these trials was below the optimal doses estimated from the dose-response trials, and the effects of split-application may become more noticeable when higher total quantities of HF are applied. Applying HF in smaller regular doses may have additional benefits, such as negating the inhibitive effects of applying large HF doses in a single event, as illustrated by the polynomial responses in shoot and root growth and reducing leaching of nutrients. Further research is required to assess the cost-benefits of single versus split HF application, and compare how these processes influence plant growth, nutrient uptake, and nutrient loss when higher quantities of HF are applied.

As is often the case with glasshouse trials testing new plant growth products, we acknowledge our study has a number of limitations that could hinder direct extrapolation of results to field conditions. We have only examined the growth of relatively young plants, using pots of limited size, and over a short duration. Additionally, we used the same potting mixes for all plants tested, and it is possible that different effects may be observed if potting mixes were modified to meet the specific requirements of each plant species. Finally, we have only considered plant growth whereas future research might consider potential qualitative responses, such as foliage protein content and nutritional value, pigment profiles, concentrations of essential oils, and resistance to pests and diseases.

Even given the limitations described above, the results provide strong evidence that HF applied as a top dressing enhances shoot and root growth in a range of vegetables, herbs, and flowering plants. Future research should extend these findings by examining other groups of plants, for example monocots or species used in mixed sward pastures, and compare effects seen in glasshouse trials with those obtained under field conditions. HexaFly currently produce approximately 500 tons of HF per annum (Alvan Hunt, personal communication, 13 September 2021) and this material would appear to be a viable soil amendment for organic food producers. Also, as it is planned to cease large-scale commercial peat extraction and production of peat-based composts in Ireland in the next decade or so [27], HF may have additional potential in the creation of organic peat-free growing media for use in the horticultural industry.

## Figures and Tables

**Table 1:** The effect of applying Hexafrass™ (HF; 1.5 g or 3 g) and chicken manure (CM; 1.5 g) on the shoot dry weight (g) of potted plants grown in a mixture of garden soil and nutrient rich compost (mean; N = 5). LSD, F and P values for each plant obtained from one-way ANOVA. Overall values calculated using all replicates and incorporating plant species in REML analysis as a random factor. Where letter codes are given, these indicate treatments separated as by the LSD as being significantly different (*p* < 0.05).

Plant	Control	HF 1.5 g	HF 3 g	CM 1.5 g	LSD	F	*p*
Basil	1.36 ^a^	2.28 ^b^	2.85 ^b^	2.22 ^ab^	0.89	4.47	0.025
Borage	1.70 ^a^	1.91 ^ab^	1.93 ^ab^	2.24 ^b^	0.39	2.33	0.064
Buckwheat	1.43	2.19	1.97	2.18	0.95	1.27	0.320
Cabbage	1.77	2.04	2.03	2.09	0.55	0.61	0.619
Celery	0.47	0.34	0.42	0.41	0.19	0.65	0.594
Chicory	1.07	1.31	1.12	1.22	0.42	0.56	0.650
Hyssop	1.27	0.86	1.21	1.01	0.52	1.20	0.342
Parsley	0.27 ^a^	0.44 ^b^	0.40 ^ab^	0.30 ^ab^	0.15	1.54	0.263
Phacelia	1.01 ^a^	1.63 ^b^	1.67 ^b^	1.38 ^ab^	0.48	3.48	0.041
Sage	0.62	0.75	0.82	0.55	0.45	0.68	0.578
Overall (dwt)	1.11 ^a^	1.37 ^b^	1.43 ^c^	1.36 ^b^	0.17	5.55	0.001
Overall Increase (%)	-	24.2	29.5	22.9			

**Table 2:** Shoot dry weight (mg; mean; n = 4#) of basil, lettuce and parsley seedlings grown in high or low nutrient potting mix and treated with 2 g HexaFrass™ (HF), chicken manure (CM) or organic fertilizer (OF). *p* values obtained from unbalanced ANOVA; treatments in each row not sharing same letter code are separated at *p* < 0.05 using LSD. # only one plant survived to harvest in this treatment.

Plant	Potting Mix	Control	HF	CM	OF	LSD	F	*p*
Basil	Low	19.3 ^a^	280.7 ^b^	203.2 ^b^	332.0 ^b^	132.5	10.6	0.002
	High	46.7 ^a^	618.0 ^b^	560.5 ^b^	433.3 ^b^	279.5	8.20	0.004
Lettuce	Low	25.3 ^a^	361.0 ^ab^	215.8 ^a^	634.7 ^b^	337.3	5.6	0.014
	High	73.0 ^a^	479.8 ^b^	372.3 ^b^	560.0 ^b^	210.6	9.9	0.002
Parsley	Low	3.2 ^a^	47.7 ^a^	33.0 ^a^	3.0 ^a#^	55.0	1.7	0.24
	High	12.5 ^a^	432.0 ^c^	232.5 ^b^	156.7 ^ab^	159.6	11.4	<0.001

**Table 3:** Estimates of parameters (± SE) for polynomial and linear regression for shoot and root dry wt (mg) and shoot:root (S:R) dry wt ratio for basil, lettuce and parsley plants treated with different quantities of HexaFrass™ (HF). Polynomial regressions were of the form *y* = *a* + *b* (HF) + *c* (HF^2^). Linear equations were of the form *y* = *a* + *b* (HF). HF_Max_ is the point estimate of HF calculated as −*b*/2*c* when the maximum value (Max) of each response variable would be obtained (see Figure 1 and Figure 2 for graphs).

Plant	Potting Mix	*y*	*a*	*b*	*c*	HF_Max_	Max
Basil	Low	Shoot	7.9 ± 45.9	175.1 ± 32.5	−14.8 ± 3.8	5.92	525.8
	Low	Root	17.5 ± 11.3	49.6 ± 8.0	−5.2 ± 0.9	4.77	135.8
	Low	S:R	1.76 ± 0.24	0.51 ± 0.06	−	−	−
	High	Shoot	101.4 ± 72.3	230.9 ± 51.2	−15.8 ± 6.0	7.31	945.0
	High	Root	47.1 ± 16.8	45.8 ± 11.9	−4.6 ± 1.4	4.98	161.1
	High	S:R	2.36 ± 0.27	0.70 ± 0.07	−	−	−
Lettuce	Low	Shoot	−23.9 ± 30.1	244.8 ± 21.3	−23.5 ± 2.5	5.21	613.6
	Low	Root	25.5 ± 9.8	59.4 ± 6.79	−7.1 ± 0.8	4.18	149.7
	Low	S:R	0.94 ± 0.39	0.98 ± 0.09	−	−	−
	High	Shoot	136.9 ± 40.3	181.0 ± 28.5	−14.3 ± 3.3	6.33	709.6
	High	Root	72.4 ± 16.6	38.6 ± 11.8	−4.0 ± 1.4	4.83	165.5
	High	S:R	1.79 ± 0.27	0.88 ± 0.19	−0.06 ± 0.02	−	−
Parsley	Low	Shoot	37.9 ± 11.4	−	−	−	−
	Low	Root	−2.4 ± 5.0	15.2 ± 4.8	−1.6 ± 0.6	4.75	33.7
	Low	S:R	6.1 ± 1.5	−	−	−	−
	High	Shoot	16.6 ± 44.9	203.3 ± 31.9	−18.6 ± 3.81	5.47	572.1
	High	Root	23.6 ± 12.4	42.9 ± 8.6	−4.4 ± 1.0	4.88	128.2
	High	S:R	1.54 ± 0.30	1.27 ± 0.21	−0.10 ± 0.02	−	−

## Data Availability

Raw data used in this study are available on request from the authors.

## Supplementary Materials

The following are available online at https://www.mdpi.com/article/10.3390/insects12110977/s1, Table S1: Days between sowing of test plants and 1st application of HexaFrass™ and chicken manure, 2nd application of HexaFrass™, and the final harvest in Trial, Table S2: p-values obtained from ANOVA examining the effects of potting mix, fertilizer treatments, and the interaction term on shoot dry matter of basil, lettuce, and parsley, Figure S3: Meta analysis using REML function in Genstat of effects on shoot dry weight of adding fertilizers to 10 different plant species grown in high organic content growing medium, Figure S4: Comparison of shoot and root growth (mg dry weight) of basil, lettuce and parsley grown in two experimental potting mixes assigned as having “Low” and “High” nutrient levels.
