# Supplementary material for: Glasshouse Evaluation of the Black Soldier Fly Waste Product HexaFrass™ as an Organic Fertilizer
Source: Insects. 2021 Oct 28;12(11):977. doi: 10.3390/insects12110977 (PMC8625123; doi:10.3390/insects12110977)

Supplementary Information

Table S1. Days between sowing of test plants and 1st application of HexaFrass™ and chicken manure, 2nd application of HexaFrass™, and the final harvest in Trial #1

Plant	Sown	1st HF application (d)	2nd HF application (d)	Harvest (d)
Basil	16/06/2021	27	40	54
Borage	16/06/2021	16	27	37
Buckwheat	16/06/2021	16	27	37
Cabbage	16/06/2021	20	28	37
Celery	16/06/2021	28	40	54
Chicory	16/06/2021	19	28	40
Hyssop	16/06/2021	27	40	54
Parsley	16/06/2021	28	40	54
Phacelia	16/06/2021	19	28	37
Sage	16/06/2021	28	40	54

Table S2. *p* -values obtained from ANOVA examining the effects of potting mix, fertilizer treatments, and the interaction term on shoot dry matter of basil, lettuce, and parsley. Potting mix factor consisted of two levels (high and low nutrients) and fertilizer treatment consisted of three levels (no fertilizer, 1 x 4 g Hexafrass™, and 4 x 1 g Hexafrass™.

Plant	Potting mix	Fertilizer treatment	Interaction term
Basil	0.002	< 0.001	0.132
Lettuce	0.905	< 0.001	0.542
Parsley	< 0.001	< 0.001	< 0.001

Figure S3. Meta analysis using REML function in Genstat of effects on shoot dry weight of adding fertilizers to 10 different plant species grown in high organic content growing medium. Treatments were (A) 1.5 g chicken manure, (B) 1.5 g HexaFrass and (C) 3g HexaFrass, per pot. Effect size for each plant estimated using Hedge's G, with 95% CIs.

A) Chicken manure (Fixed effect P = 0.019, Random effect P = 0.085)

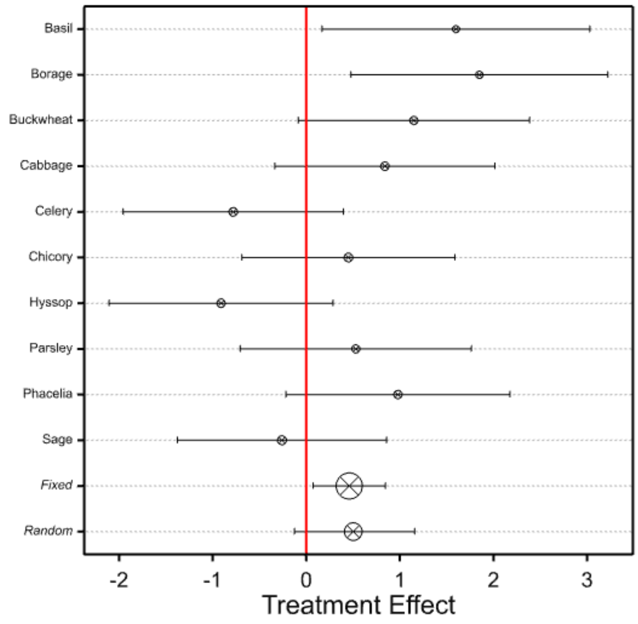

B) HexaFrass 1.5 g (Fixed effect P = 0.001, Random effect P = 0.067)

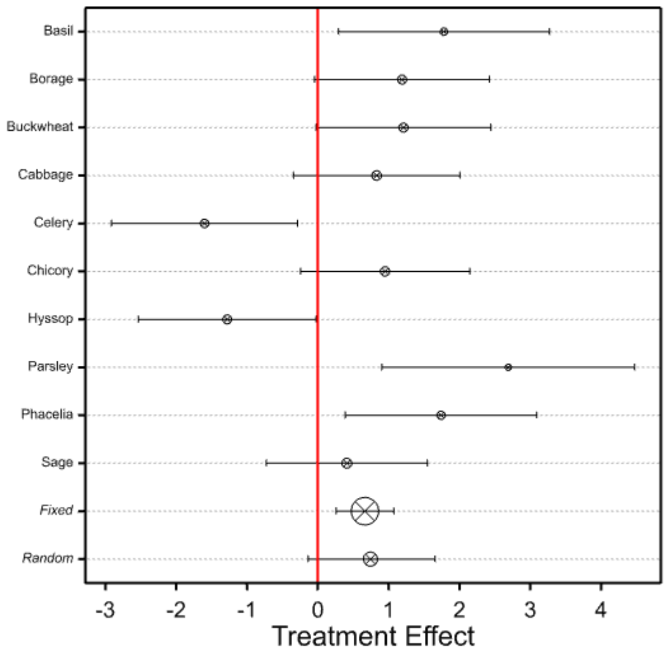

C) HexaFrass 3 g (Fixed effect $P < 0.001$, Random effect $P = 0.006$)

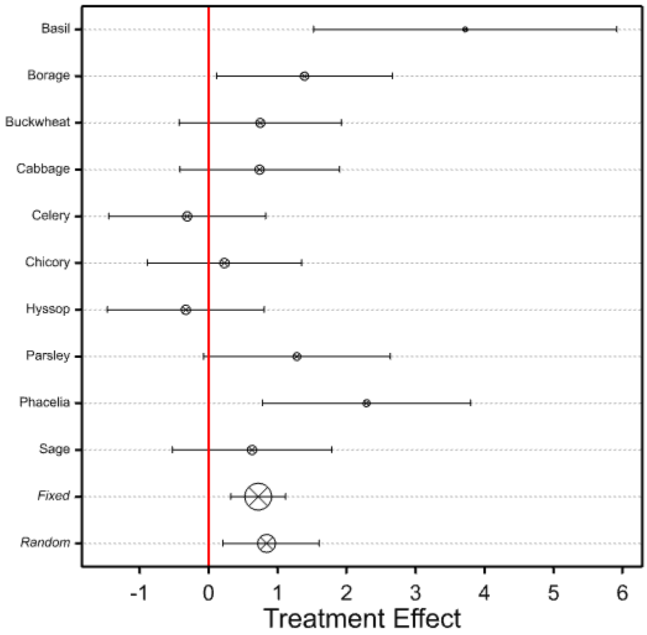

Figure S4. Comparison of shoot and root growth (mg dry weight) of basil, lettuce and parsley grown in two experimental potting mixes assigned as having “Low” and “High” nutrient levels. Overall comparisons using REML models and assigning plant species as a random factor indicated significant differences between potting mixes for both shoots (p = 0.002) and roots (p < 0.001).

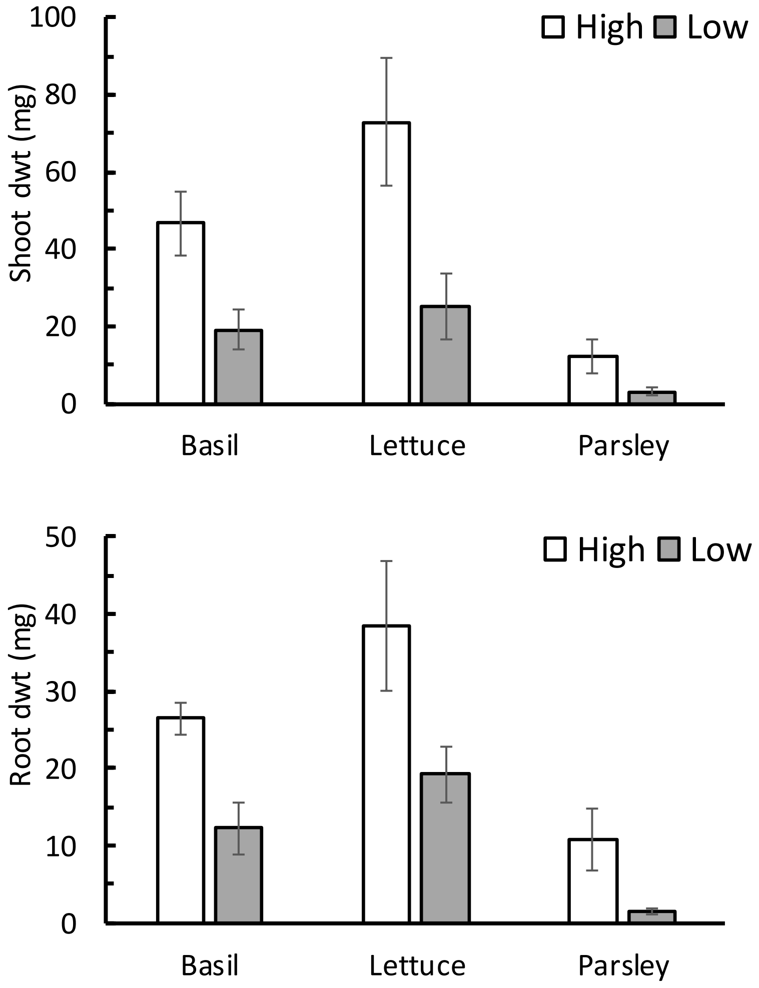

Supplement: Supplementary file 1 [file insects-12-00977-s001.zip › insects-1418501-SUPPLEMENTARY.pdf]
